# Influence of feeder cells on transcriptomic analysis of pluripotent stem cells

**DOI:** 10.1111/cpr.13189

**Published:** 2022-01-21

**Authors:** Haifeng Wan, Rui Fu, Man Tong, Yukai Wang, Libin Wang, Siqi Wang, Ying Zhang, Wei Li, Xiu‐Jie Wang, Guihai Feng

**Affiliations:** ^1^ State Key Laboratory of Stem Cell and Reproductive Biology Institute of Zoology Chinese Academy of Sciences Beijing China; ^2^ Institute for Stem Cell and Regenerative Medicine Chinese Academy of Sciences Beijing China; ^3^ Beijing Institute for Stem Cell and Regenerative Medicine Beijing China; ^4^ Key Laboratory of Genetic Network Biology Institute of Genetics and Developmental Biology Innovation Academy of Seed Design Chinese Academy of Sciences Beijing China; ^5^ National Stem Cell Resource Center, Chinese Academy of Sciences Beijing China; ^6^ University of Chinese Academy of Sciences Beijing China; ^7^ Present address: Shanghai Cell Therapy Group Shanghai China

## Abstract

**Objectives:**

Human pluripotent stem cells (hPSCs) are of great importance in both scientific research and regenerative medicine. The most classic and widely used culture method for hPSCs is co‐culture with feeder cells, usually mouse embryonic fibroblasts. However, whether these feeder cell residues can affect the transcriptomic data analysis of hPSCs, especially gene or miRNA expression quantification, is still largely unknown.

**Methods and Results:**

In this study, reanalysis of published mRNA‐Seq and miRNA‐Seq data sets revealed the existence of feeder cell‐derived reads in the hPSC transcriptomic samples. We identified potentially influenced human genes and miRNAs due to misalignment of sequencing fragments affected by mouse feeder cells. Furthermore, we developed an optimized miRNA analysis pipeline to avoid quantification bias from different miRNA isoforms in the same family. Finally, by comparing the levels of feeder cell residues in hPSC samples isolated by different methods, we found that fluorescence‐activated cell sorting and adhesion methods were more effective in feeder cell removal than the gradient centrifugation method.

**Conclusions:**

Collectively, our results demonstrate that feeder cell residues affect the transcriptomic data analysis of hPSCs. To minimize the impact of feeder cell contamination in hPSC samples, we provide solutions for both data analysis and sample preparation.

## Graphical abstract

The existence of feeder cell‐derived reads in the hPSC transcriptomic samples. Some human genes and miRNAs are potentially influenced by reads from feeder cells. FACS and adhesion methods are effective in feeder cell removal.

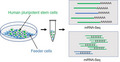

## 1 INTRODUCTION

Human pluripotent stem cells (hPSCs) are capable of self‐renewal and differentiating into three germ layers, exhibiting great potential in regenerative studies and clinical therapies.^1^, ^2^ Often, hPSCs are cultured on plates seeded with feeder cells, which provide growth factors and extracellular matrix necessary for the pluripotency maintenance of these cells.^3^ Feeder cells used in hPSC co‐culture systems are commonly produced from mouse embryonic fibroblasts (MEFs) pre‐treated with either mitomycin C (MMC) or gamma‐ray irradiation. These treatments have no influence on cell viability, but can inhibit MEF mitosis and proliferation.^4^ The feeder system is well established for all cultured hPSCs, including embryonic stem cells (ESCs) and induced pluripotent stem cells. Although feeder‐free culture methods have been developed, this co‐culture system is highly recommended for fragile cell types, such as naïve or ground state hPSCs.^5^, ^6^, ^7^

Experimentally, isolation of hPSCs from feeder cells relies on gradient centrifugation or different adhesion abilities after cell re‐seeding. However, complete removal of feeder cells is almost impossible in both methods, suggesting that feeder cell contamination might be present in the isolated hPSC samples. Common transcriptomic data analysis pipelines map sequencing data only to the target genome (the human genome for hPSCs); therefore, the impact of feeder cell residues on hPSC transcriptomic data analysis is worthy of investigation. In a recent study, Stirparo et al. found that feeder cell residues in naïve hPSCs led to erroneous cancer‐related SNP calls.^8^ However, whether feeder cell contamination has an impact on gene expression quantification, especially for miRNAs, in human cells is unknown, and the method to remove feeder cell residues in hESC sample preparation has not been demonstrated.

In this study, we focused on the influence of feeder cell residues on the analysis results of hPSC transcriptome profiles. Reanalysis of published mRNA‐Seq and miRNA‐Seq data confirmed the existence of feeder cell contamination in hESC samples harvested from the feeder system. We further identified potential genes or miRNAs that were influenced by feeder cell residues. To better analyse the miRNA‐Seq data, we redesigned the miRNA analysis pipeline and further identified feeder cell‐specific miRNA markers. In addition, we compared the levels of feeder cell residues in hESC samples isolated by different separating strategies, demonstrating that cell sorting is a better method to reduce feeder cell contamination in hPSC samples.

## 2 MATERIALS AND METHODS

### 2.1 Preparation of feeder cells

Mouse embryonic fibroblasts (MEFs) were cultured to reach 90–95% confluence. The MEFs were then treated with MMC or gamma irradiation with Cobalt 60 (Co60). For the MMC treatment, MMC was added to the culture medium at a final concentration of 10 µg/mL. Cells were cultured for 2.5 h at 37°C with 5% CO_2_, and then washed with phosphate buffered saline (PBS) three times to completely remove MMC. For the gamma irradiation treatment, MEFs were inactivated by gamma radiation at 30 Gy. Following treatments, MEFs were trypsinized and collected by centrifugation at 200 *g* for 5 min. The collected feeder cells were counted and seeded in tissue culture dishes at 1 × 10^5^ cells per 35‐mm dish.

### 2.2 Cell culture

Human embryonic stem cell (hESC) line H9 was cultured in both feeder and feeder‐free systems. In the feeder system, feeders were pre‐coated one day before H9 was passaged. Cells were cultured in 20% KOSR medium (DF12 basal medium with 20% KOSR and 10 ng/mL FGF2) and digested into clusters using collagenase IV. For the feeder‐free system, H9 cells were cultured in Essential 8 (E8, Gibco) medium and digested into clusters using 0.5‐mM EDTA. In both systems, H9 was maintained at 37°C with 5% CO_2_.

Early passage (passage 12) hESC line Q‐CTS‐hESC‐2 (Q2) was obtained from the National Stem Cell Resource Center (Institute of Zoology, Chinese Academy of Sciences). The cell line was derived as described previously.^9^ Cells were cultured in a feeder‐free system at 37°C and 5% CO_2_. Cells were seeded in vitronectin‐coated plates and maintained in E8 medium. TrypLE was used to digest cells into clusters or single cells every 4 days.

### 2.3 H9 cells collection

The H9 cells cultured in the feeder system were collected using three different methods: (1) fluorescence‐activated cell sorting (FACS): the cells were digested into single cells using accutase (Gibco) and further stained with PE Mouse anti‐Human TRA‐1‐60 Antigen (BD bioscience, 560884). The PE‐positive cells were collected on a Beckman MoFlow XDP II (Beckman), whereas the cells stained with or without antibody staining served as the controls; (2) gradient centrifugation: the cells were digested into single cells using accutase and the mixture was centrifuged for 30–60 s at 10 *g*. The supernatant, containing the H9 cells, was collected gently; (3) different adhesion times: the cells were digested into single cells using accutase, and the mixture was seeded in dishes pre‐coated with gelatine. Feeder cells usually adhere to the dish faster than human embryonic stem cells. Approximately 15 min later, the cell supernatant was collected for further analysis.

### 2.4 mRNA‐Seq data reanalysis

The raw data listed in Table S1 were downloaded from the SRA database.^10^ Data were further transferred into FASTQ format using FASTQ‐dump (version 2.8.0). The raw reads were cleaned using trim_galore (https://github.com/FelixKrueger/TrimGalore) with default settings; reads with lengths longer than 50 nt were retained. The clean reads were aligned to the human (hg38) and mouse (mm10) genomes using Hisat2 (version 2.1.0) with default settings.^11^ The reads uniquely mapped to either the hg38 or mm10 genome. Mouse‐specific reads were used for gene quantification analysis using StringTie (version 2.1.4).^12^ The mixing ratio of reads derived from feeder cells was calculated as mouse‐specific read counts divided by the sum of mouse‐ and human‐specific read counts. The genes in each mouse embryonic stem cells (mESCs) sample that showed more than a 20‐fold increase compared to those in mouse hepatocyte and MEF samples, were defined as mESC‐specific marker genes. The same criterion was used to define MEF‐ or hepatocyte‐specific genes. All genes with no less 1 fragment per kilobase of transcript per million reads mapped (FPKM), in at least one sample, were used for sample correlation analysis. The heatmaply package was used to produce a correlation heatmap.^13^ The potentially influenced human genes were identified using the feeder‐free cultured MEF and hESC samples. The reads of MEF and hESC samples were mapped to hg38 using Hisat2 with default settings, and only reads with unique genome locations were used for quantification analysis. The number of reads mapped to each annotated human gene was calculated using htseq‐count (version 0.13.5).^14^ This was then normalized to RPM (reads per million reads) using the total number of samples. The genes with a two‐fold increase in MEF samples, as compared to those in hESC samples, were identified as potentially influenced genes (Table S2). These genes are shown in a heatmap produced by the heatmap.2 function in R (version 3.5.1).

### 2.5 miRNA sequencing and analysis

#### 2.5.1 miRNA clustering

The mature miRNA annotations for humans and mice in miRBase (version 22) were used for the read annotation analyses.^15^ The mature miRNAs were mapped to both the hg38 and mm10 genomes using Burrows‐Wheeler Aligner (BWA) in “aln” mode.^16^ All the mapped hits were kept with no more than 2 differences (parameters: ‐n 2 ‐N ‐k 2 in aln mode and the ‐n was set to 90,000,000 in same mode) or 3 mismatches without gaps (parameters: ‐n 3 ‐N ‐k 3 ‐o 0 in aln mode and the ‐n was set to 90,000,000 in the same mode). All hits in the two settings were merged. The miRNAs with overlapping genome location (overlap >90%) were then merged as a single miRNA cluster. If a mouse miRNA mapped to hg38 and showed an overlap of above 90% with a human miRNA, both the mouse and human miRNAs were defined as being conserved. For example, if the mouse miRNA, miR‐A, mapped to mm10 at the same genome location as miR‐B within three mismatches; also mapped to hg38; and overlapped human miR‐C and miR‐D, then miR‐A/B__miR‐C/D was defined as a conserved cluster. However, if the mouse miRNA could be mapped to hg38 but showed no overlap with known miRNAs, miR‐A__NA was defined as a mouse‐specific Type‐B cluster. Whereas if the reads could not be mapped to hg38, miR‐A__NA was defined as a mouse‐specific Type‐A cluster. Using these same principles, human‐specific miRNA clusters were also defined.

#### 2.5.2 miRNA quantification

Total RNA was extracted using an RNA isolation kit (Ambion) according to the manufacturer's instructions. Small RNA fractions (18–50 nt) were isolated from total RNA by PAGE and ligated to a pair of adaptors at the 5′ and 3′ ends. Small RNA molecules were converted to cDNA and amplified by RT‐PCR using adaptor primers. Purified DNA was directly used for cluster generation and sequencing analysis using an Illumina HiSeq 4000 or HiSeq X‐Ten platform.

Apart from the data from our lab, the public small RNA‐Seq data, downloaded from the SRA database, was also used (Table S1). All published hESC samples were clearly described as cultured in an MEF feeder system according to the methods of the original publication.

All miRNA‐Seq samples were filtered and trimmed using Cutadapt (10.14806/ej.17.1.200) and the custom 5′ and 3′ adaptor sequences, described in the original methods, were used. Only clean reads with lengths between 18–30 nt were retained. The hg38 and mm10 reference genomes were used for the mapping analysis.

Similar to the mature miRNA analysis, the sequencing reads were mapped to both genomes by BWA in “aln” mode.^16^ All the mapped hits were kept with no more than 2 differences (parameters: ‐n 2 ‐N ‐k 2 in aln mode and the ‐n was set to 90,000,000 in the same mode). Reads with at least one hit overlapping the genome location of a defined miRNA cluster were used for miRNA cluster quantification. Last, each miRNA cluster abundance was normalized by RPM (Reads Per Million Mapped Reads). For the marker miRNA identification, the criterion for Type‐B and conserved miRNAs was defined as more than 10 RPM in feeder cells and less than 1 RPM in human ESC samples cultured in feeder‐free system. Type‐A miRNAs within more than 1 RPM in feeder cells were also selected as marker miRNAs.

All clean reads in FASTQ format with more than a 90% overlap with mature miRNAs were considered as reads produced from the mature miRNAs. All target mature RNAs that overlapped with different targets, by one read, were clustered. This clustering process was repeated according to the target miRNAs. For example, if read M was mapped to mouse miR‐A and miR‐B, while read N was mapped to mouse miR‐B and miR‐C, these two reads could be mapped to human miR‐D and miR‐E. The miR‐A/B/C__miR‐D/E cluster was defined as conserved with two reads, for use in the expression level analysis. If the reads could be mapped to hg38 but did not overlap with known mature miRNAs, then miR‐A/B/C__NA was defined as a mouse‐specific Type‐B cluster. Finally, if the reads could not be mapped to hg38, miR‐A/B/C__NA was defined as a mouse‐specific Type‐A cluster. Human‐specific miRNA clusters were also defined this way. Potentially influenced human RNAs were defined as conserved miRNA clusters with a 10‐fold increase in cells cultured in the feeder system, compared to those in cell cultured in the feeder‐free system.

Principal component analysis (PCA) and heatmaps were performed with the prcomp and heatmap.2 functions in R (version 3.5.1). Violin plots were produced using ggplot2 in R. The distribution of miRNAs along chromosomes was shown by circos.^17^

## 3 RESULTS

### 3.1 Reads derived from feeder cells are stably detected in published hPSC mRNA‐Seq data sets

**FIGURE 1:**
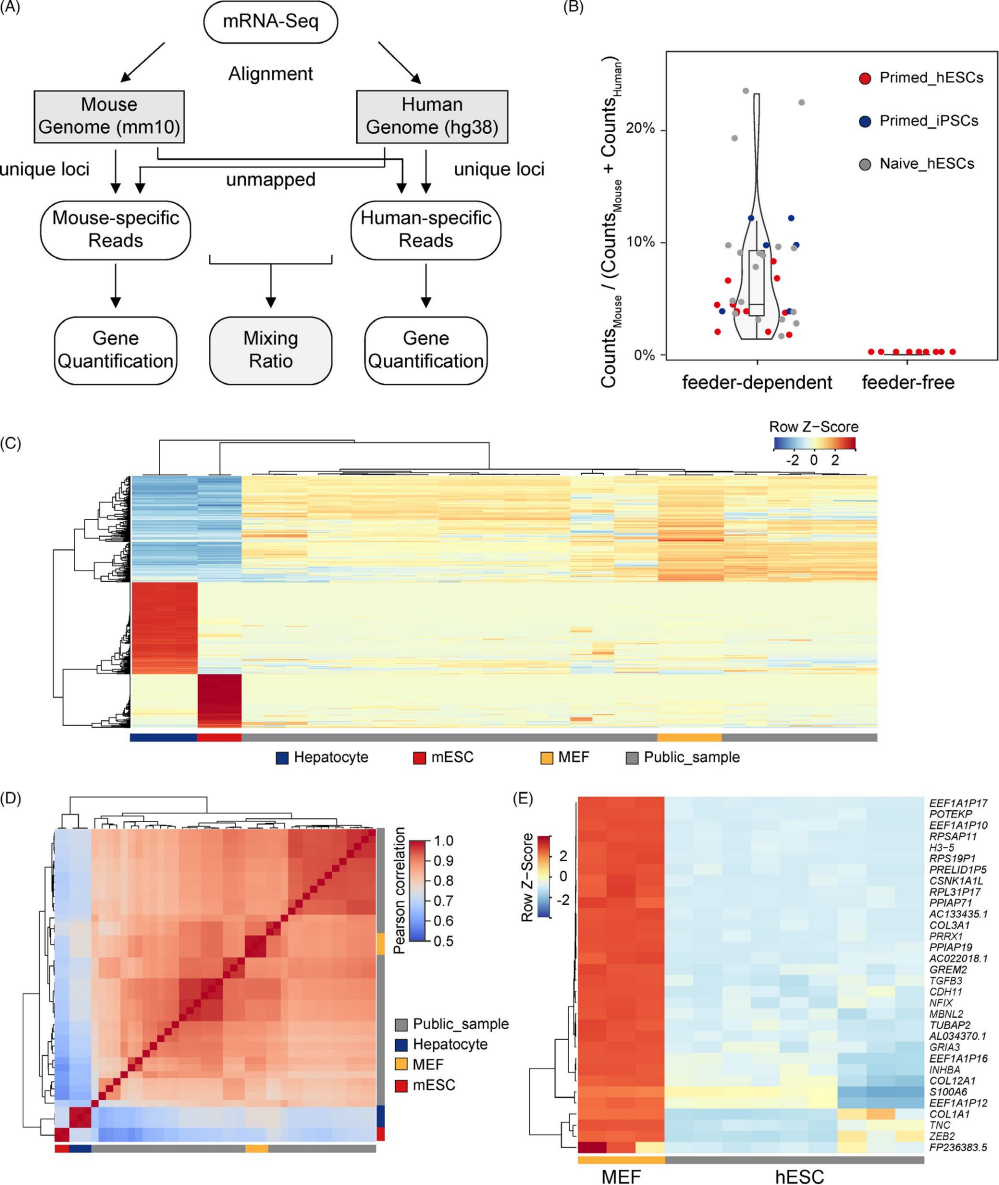
Evaluation of feeder cell residues in hPSC mRNA‐Seq data sets. (A) Strategy for identification of species‐specific reads in mRNA‐Seq. (B) Ratio of mouse‐derived reads in mRNA‐Seq data sets of hPSCs maintained in different cultured systems. (C) Expression pattern of mESC, hepatocyte and MEF marker genes in mouse‐specific read sets separated from hPSC mRNA‐Seq data sets. (D) Correlation analysis for the whole genome‐wide gene expression pattern of mouse‐specific read sets separated from hPSC mRNA‐Seq of the ESC, hepatocyte and MEF data sets. (E) Human genes whose expression levels might be influenced by reads derived from residual mouse feeder cells

To evaluate the levels of feeder cell residues in hPSC samples, published mRNA‐Seq data sets from the SRA database were used to identify mouse feeder cell‐derived reads. We mainly focused on analysis of reads with longer lengths, since serving as the most common sequencing data type in transcriptomic data analysis, these reads in mRNA‐Seq were easier to track their species origin. We reanalysed 36 data sets of hPSCs cultured in an MEF‐derived feeder‐dependent system and 9 samples cultured in a feeder‐free system (Table S1). We used a mixing ratio to evaluate the proportion of mouse‐derived sequences to the sum of mouse‐derived and human‐derived sequences. To ensure the accuracy of analysis, only the reads with mutually exclusive alignment to the human or mouse genomes were retained (Figure 1A). Unexpectedly, the mixing ratio was very high (median = 4.5%, maximum = 23.3%, minimum = 1.4%) in hPSC samples cultured under the feeder‐dependent conditions, whereas samples from the feeder‐free culture system rarely had mouse‐derived sequences (median = 9.12e‐5; Figure 1B). Subsequently, we evaluated whether these mouse‐specific reads separated from hESC samples were derived from feeder cell residues or nonspecific sample contamination. Mouse‐specific reads separated from hESC samples as well as reads from MEFs, mESCs and mouse hepatocyte samples were used for gene expression quantification. Only marker genes in MEFs, the source of feeder cells, had a similar expression pattern to genes calculated by these mouse‐specific reads (Figure 1C). Additionally, the genome‐wide gene expression pattern of the mouse‐specific reads separated from hESC samples displayed a more similar pattern to MEFs compared to those from mESC or hepatocyte samples (Figure 1D). We, next, mapped MEF reads to the human genome and identified human genes whose expression levels were influenced by misalignment caused by sequence similarity. We compared the gene expression in hESCs cultured in the feeder‐free system to their false positive expression from MEF‐derived reads mapping to the human genome (Table S2). We found 125 human genes that displayed at least a two‐fold increase in MEF‐derived reads, and 32 of 125 genes showed more than a five‐fold increase (Figure 1E), suggesting that more attention should be paid to these human genes because their increased expression might be mistaken for MEF contamination.

### 3.2 Feeder cell‐specific miRNAs are identified by an optimized miRNA analysis pipeline

**FIGURE 2:**
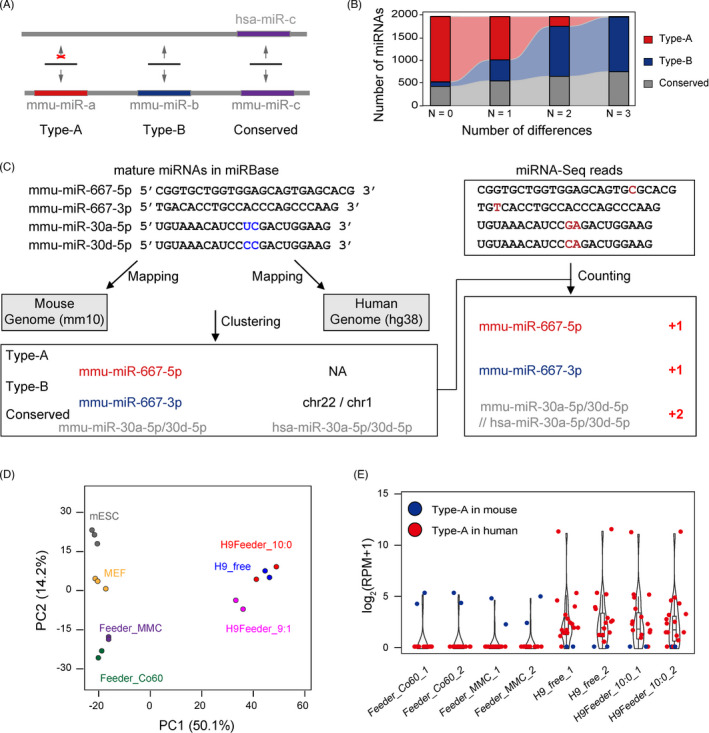
Identification of feeder cell‐specific miRNAs. (A) Schematic diagram showing the definition of miRNA types based on the alignment of mouse miRNAs to the human genome. (B) Distribution of mouse miRNA types that were mapped to the human genome with a specific distance setting. (C) Strategy for the annotation of miRNA clustering and miRNA quantification. (D) Principal component analysis (PCA) of miRNA expression profiles based on the established counting strategy. The percentages of variation explained by the principal components are marked in brackets. H9Feeder_9:1 means mixed H9hESCs and feeder cells with a 9:1 ratio. (E) Expression level of Type‐A miRNAs or miRNA clusters in feeder cells, H9hESCs cultured in a feeder‐free system, and H9hESCs cultured in a feeder system isolated by FACS. The miRNA expression level (RPM) was normalized by log2

To better examine the presence of feeder cell contamination in hPSC samples harvested from the feeder system, we attempted to identify mouse feeder‐specific miRNAs from hPSC miRNA‐Seq data. We aligned all mouse mature miRNAs in miRBase to the human genome, and further classified them into three types: Type‐A, which had no matching sequences in the human genome; Type‐B, which could be aligned to the human genome but did not overlap with annotated human miRNAs; and Conserved, which were conserved miRNAs that could be aligned to the human genome and also overlapped with human miRNA loci (Figure 2A). Since Type‐A miRNAs cannot be mapped to the human genome, they should be the best choice to distinguish the species origin of miRNAs. Notably, the parameter of allowed differences in alignment, including mismatches and gaps, in the custom miRNA analysis pipeline can affect the number of Type‐A miRNAs. The percentage of mouse Type‐A miRNAs dramatically decreased from 73.3% (1442) to 0.6% (12) when the number of allowed mismatches was defined from 0 to 3 (Figure 2B). A similar trend was observed when human miRNAs were mapped to the mouse genome (Figure S1A). Additionally, the sequences of different mature miRNAs from the same family show high similarities,^18^ leading to the failure of miRNA quantification. To address these issues, we developed a new strategy for quantitative miRNA‐Seq analysis (Figure 2C).

All mature human and mouse miRNAs from miRBase were merged and simultaneously mapped to the human and mouse genomes. The miRNAs were clustered if they mapped to the same genome location with no more than three differences allowed (three mismatches or two mismatches with a gap open, allowed in BWA settings). In total, 3490 miRNA clusters were constructed, including 74 Type‐A (32 from mice and 42 from humans), 2916 Type‐B, and 500 conserved miRNA clusters (Tables 1 and S3). In our strategy, the human and mouse miRNAs were mixed together in our pipeline and further quantified. Each miRNA cluster, rather than a single miRNA, served as a unit for quantification analysis, avoiding the read misalignment of miRNAs with similar sequences (Figure 2C). The effectiveness of our optimized strategy in cell type classification was confirmed by unsupervised clustering and principal component analysis (PCA), although some information was lost (Figures 2D and S1B). Using PCA, the miRNA profiles of feeder cells inactivated by either MMC treatment or gamma‐ray irradiation displayed a high similarity to the untreated MEFs (Figures 2D, S1B and C). The miRNA data of hESCs obtained from either the feeder‐free system or isolated from the feeder system by FACS displayed similar expression patterns (Figure S1D). However, the miRNA profiles of samples of which hESCs and feeder cells were mixed at a 9:1 ratio were deflected to mouse‐derived cells (Figure 2D). Using our optimized strategy, we identified 2 mouse and 16 human Type‐A miRNAs from mouse feeder cells and hESC samples, respectively (Figure 2E and Table 1), indicating that these Type‐A miRNAs could serve as feeder cell‐specific markers. We found similar results for most, but not all, Type‐B miRNAs (Figure S1E), and we speculate that the misalignments might be caused by some Type‐B miRNAs overlapping with other small RNA genome loci, such as rRNAs, snRNAs or snoRNAs (Table S3).

**TABLE 1:** Type‐A miRNAs of the annotated mouse and human miRNAs in miRbase (version 22)

Species	Type‐A miRNAs		
Mouse	*mmu‐miR‐1932* *mmu‐miR‐1966‐5p* *mmu‐miR‐290a‐3p* ***mmu‐miR‐3057‐5p*** *mmu‐miR‐3066‐3p* *mmu‐miR‐3104‐5p* *mmu‐miR‐453* *mmu‐miR‐468‐5p* *mmu‐miR‐489‐5p* *mmu‐miR‐5135* *mmu‐miR‐6546‐5p*	*mmu‐miR‐5625‐3p* *mmu‐miR‐6351* ***mmu‐miR‐667‐5p*** *mmu‐miR‐6896‐5p* *mmu‐miR‐6900‐5p* *mmu‐miR‐6970‐5p* *mmu‐miR‐6977‐5p* *mmu‐miR‐7004‐5p* *mmu‐miR‐7005‐3p* *mmu‐miR‐7032‐5p* *mmu‐miR‐7037‐5p*	*mmu‐miR‐7081‐5p* *mmu‐miR‐7115‐5p* *mmu‐miR‐7222‐5p* *mmu‐miR‐7226‐5p* *mmu‐miR‐7236‐5p* *mmu‐miR‐741‐5p* *mmu‐miR‐7659‐3p* *mmu‐miR‐7677‐5p* *mmu‐miR‐6386* *mmu‐miR‐7006‐5p*
*Human*	*hsa‐miR‐1183* *hsa‐miR‐12119* *hsa‐miR‐12127* *hsa‐miR‐12128* ***hsa‐miR‐1272*** ***hsa‐miR‐1292‐5p*** ***hsa‐miR‐1307‐3p*** ***hsa‐miR‐2277‐5p*** ***hsa‐miR‐3143*** ***hsa‐miR‐3180‐5p*** *hsa‐miR‐3616‐3p* *hsa‐miR‐3690* *hsa‐miR‐3977* ***hsa‐miR‐4518***	*hsa‐miR‐4641* ***hsa‐miR‐4664‐5p*** *hsa‐miR‐4665‐3p* *hsa‐miR‐4685‐5p* *hsa‐miR‐4706* ***hsa‐miR‐4745‐3p*** ***hsa‐miR‐4754*** *hsa‐miR‐5188* ***hsa‐miR‐573*** ***hsa‐miR‐579‐5p*** ***hsa‐miR‐597‐3p*** *hsa‐miR‐6081* ***hsa‐miR‐636*** ***hsa‐miR‐8074***	*hsa‐miR‐637* *hsa‐miR‐658* *hsa‐miR‐6765‐5p* *hsa‐miR‐6766‐5p* *hsa‐miR‐6768‐3p* ***hsa‐miR‐6770‐5p*** *hsa‐miR‐6831‐5p* *hsa‐miR‐6840‐5p* *hsa‐miR‐7107‐3p* *hsa‐miR‐7161‐3p* ***hsa‐miR‐7706*** *hsa‐miR‐8053* *hsa‐miR‐8069* *hsa‐miR‐8080*

Note

The expressed miRNAs in feeder cells or hESCs are marked in bold.

### 3.3 Feeder cell‐specific miRNAs are detected in majority of published hESC miRNA‐Seq samples cultured in feeder system

**FIGURE 3:**
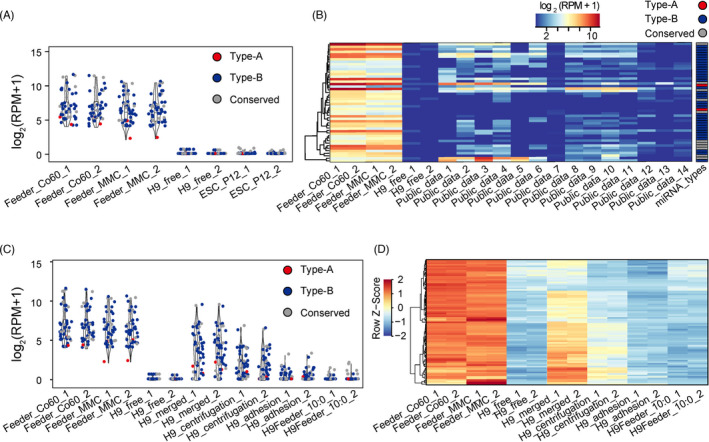
Assessment of feeder cell residues in hPSC miRNA‐Seq data sets. (A) Specifically expressed miRNAs or miRNA clusters in feeder cells compared to low and high passaged hECSs. The miRNA expression level (RPM) was normalized by log2. (B) Heatmap showing the expression levels of marker miRNAs in published hESCs cultured in a feeder system. The marker types are listed on the top‐right and the miRNAs are listed in Table S2. (C) Specific miRNA residual levels of feeder cells in H9 hESCs isolated by different methods. (D) Conserved miRNAs between human and mouse whose expression levels might be influenced by reads derived from residual mouse feeder cells

Both early‐passage and long‐term cultured hESCs were harvested and sequenced to attenuate the expression bias caused by differences in cell passages.^19^ Using rigorous thresholds, 48 feeder cell‐specific miRNA clusters (2 Type‐A, 35 Type‐B and 11 Conserved) were identified and used to determine feeder cell contamination in hESC samples (Figure 3A and Table S4). Interestingly, although feeder cell‐specific miRNA markers were distributed across the whole genome, they were preferentially located within the Dlk1‐Dio3 and miR‐344 family regions, which could be investigated in future (Figure S2 and Table S4).

Using these feeder cell‐specific miRNAs, we further evaluated whether feeder cell contamination existed in the 14 published miRNA‐Seq data sets derived from feeder‐cultured hESCs (Table S1). Consistent with the mRNA‐Seq results, the majority of hESC samples cultured in feeder systems showed feeder cell contamination (Figures 3B and S3A). For instance, mmu‐miR‐667‐5p, a mouse‐specific Type‐A miRNA with low basal in feeder cells (Figure 3A), was detected with at least one read in seven hESC miRNA‐Seq samples, indicating the existence of feeder cell residues in these published hESC samples (Figures 3B and S3A).

Next, we assessed the levels of feeder cell residues in hESCs isolated by cell sorting, gradient centrifugation and the adhesion method. Compared to the cells cultured in the feeder‐free system, hESC samples isolated by density gradient centrifugation showed significantly higher levels of miRNAs derived from feeder cells, whereas the hESCs sorted by either FACS, TRA‐1–60 or the adhesion method contained low levels of feeder cell residues (Figure 3C). Similar to mRNA‐Seq analysis, we identified 85 conserved miRNA clusters between hESCs and mouse feeder cells, whose expression levels had at least a 10‐fold increase in mouse feeder cells compared to those in hESCs, suggesting their potential to interfere with the analytical results of hESC transcriptomes (Figures 3D, S3B and Table S2). Therefore, more attention should be paid to these miRNAs when differential expression analysis is performed with other cell types.

## 4 DISCUSSION

The current study demonstrates that feeder cell contamination can influence hPSC transcriptomic data analysis, which is a widespread but easily ignored problem. As described in a recent paper,^8^ apart from the potential confounding effects on transcriptomic quantification, feeder cell residues could also influence nucleotide variation calling due to similar site divergence between mouse and human regions. Except for SNV calling, RNA editing by ADARs is often found in ESCs with a low editing ratio.^20^, ^21^, ^22^ When analysing the RNA editing ratio in ESCs cultured in feeder systems, more attention should be paid to exclude the false positives introduced by feeder cell residues.

In clinical applications, hPSCs are often cultured in xeno‐free systems.^23^, ^24^ Human‐derived feeder cells were used for PSC culturing. Under such conditions, the feeder cell residue levels could be evaluated by feeder cell‐specific SNPs or specific gene expression.

Multiple factors in miRNA‐Seq analyses, including initial nucleotide concentration, library construction protocols, the number of PCR cycles, sequencing devices and even barcode sequences, could influence miRNA quantification.^25^ Therefore, to quantify the levels of feeder cell residues in miRNA‐Seq, it is necessary to establish robust models that balance these factors to evaluate the residual ratio. As an alternative option, mRNA‐Seq or low‐coverage whole‐genome sequencing, which have longer read lengths, could be performed together with smRNA‐Seq for residual ratio evaluation.

Notably, the mRNA‐based strategy shown in this study can also be used to detect other cross‐contaminations, apart from feeder cell contamination, which is a common issue in cell culturing.^26^, ^27^ These cross‐contaminations can include parasitic microorganism contamination, caused by mycoplasma or bacterial contamination, and unwanted cell contamination due to mislabelling or misoperation in the culture process. Finally, the application of the mRNA‐Seq analysis pipeline can not only be used to quantify feeder cell residues, but can also be used to assess multiple types of cell culture contaminations.

## CONFLICT OF INTEREST

The authors declare that they have no competing interests.

## AUTHOR CONTRIBUTIONS

G.F. and X.‐J.W. conceived and designed the study. X.‐J.W., and W.L. supervised the project. H.W., R.F. and M.T. performed the experiments. Y.W. and L.W. were involved in the methodology. G.F. and H.W. analysed the data and wrote the manuscript. S.W. and Y.Z. were involved in the manuscript preparation.

## Supporting information

Supplementary Material

Table S2

Table S3

## Data Availability

The sequencing data were deposited in the Genome Sequence Archive (GSA) and GSA‐Human of the Beijing Institute of Genomics, Chinese Academy of Sciences (http://gsa.big.ac.cn/). The accession numbers for the sequencing data reported in this study are CRA005711 and HRA001729.
