# Supplementary material for: Influence of feeder cells on transcriptomic analysis of pluripotent stem cells
Source: Cell Prolif. 2022 Jan 21;55(2):e13189. doi: 10.1111/cpr.13189 (PMC8828260; doi:10.1111/cpr.13189)
Supplement: Supplementary file 1 — Supplementary Material [file CPR-55-e13189-s001.docx]

**Supplemental Information**


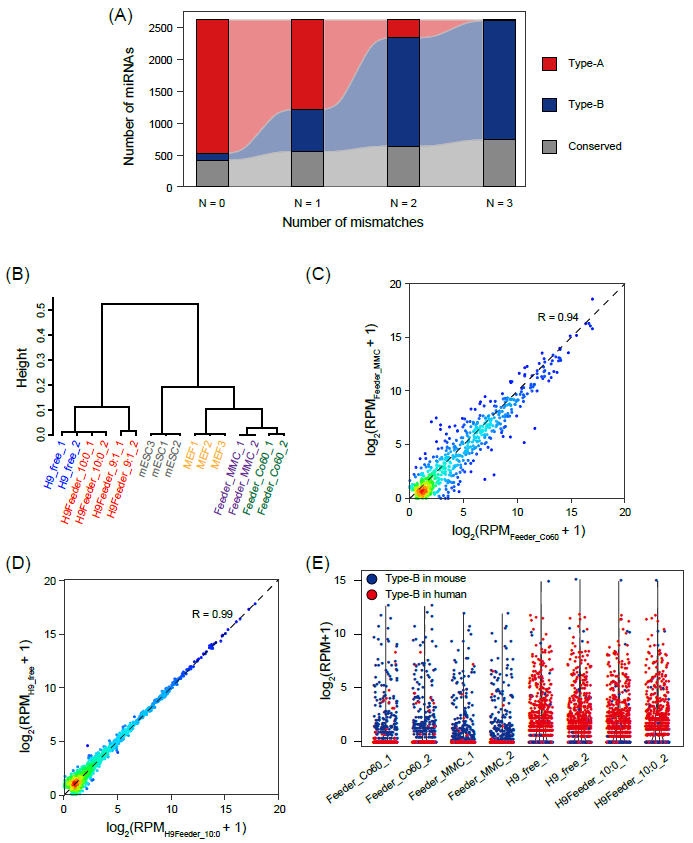


Figure S1. **Analysis of miRNA types and their expression patterns**.

(A) Distribution of human miRNA types mapped to the mouse genome with a specific edit distance setting. (B) Unsupervised hierarchical clustering of miRNA expression profiles based on the established counting strategy. (C) Scatter plot of the miRNA expression levels of the feeder cells treated with Co60 or mitomycin C. Average expression was calculated with two replicates. The miRNA expression level (RPM) was normalized by log2. The Pearson correlation coefficient is shown. (D) Scatter plot of the miRNA expression levels of H9 hESCs cultured with different systems. The average expression was calculated with two replicates. The miRNA expression level (RPM) was normalized by log2. The Pearson correlation coefficient is shown. (E) Expression level of Type B miRNAs or miRNA clusters in feeder cells, H9 hESCs cultured in a feeder-free system, and H9 hESCs cultured in a feeder system isolated by FACS. The miRNA expression level (RPM) was normalized by log2.


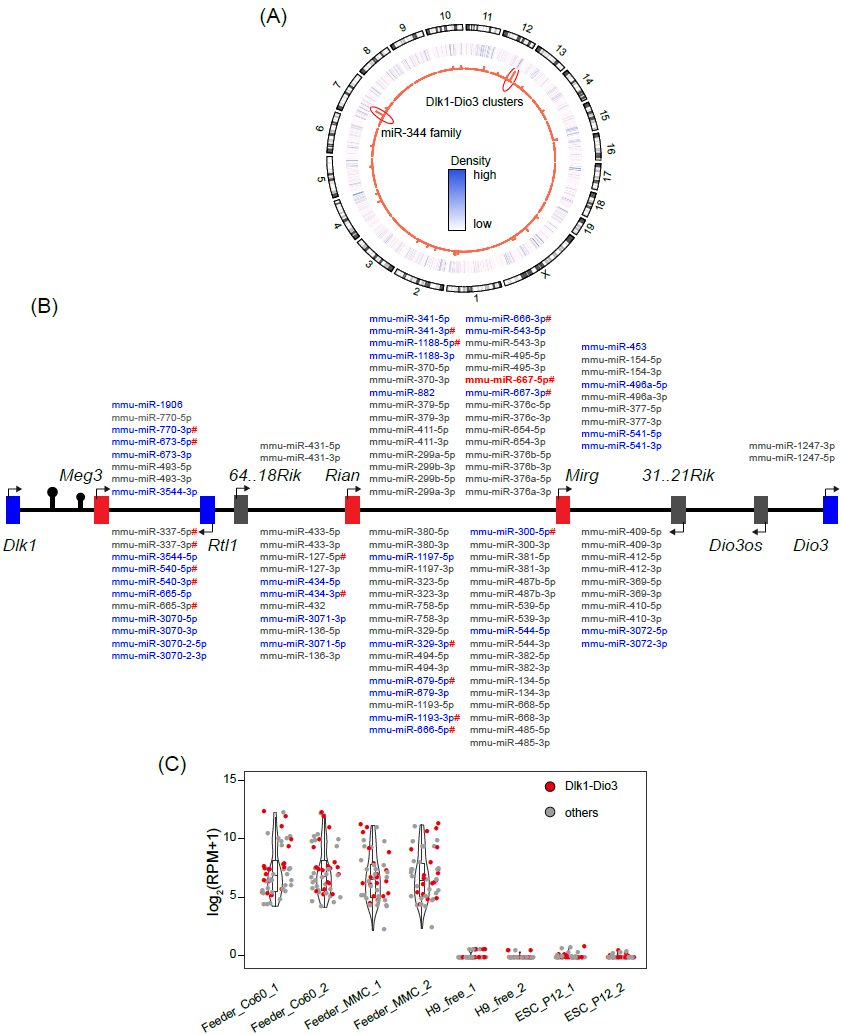


Figure S2. **Analysis of miRNAs expression in the Dlk1-Dio3 region**.

(A) Circos plot of the marker miRNA distribution along the mouse genome. The outer layer shows the chromosome karyotype, the middle layer shows all the annotated miRNAs distributed along the genome. The inner histogram shows the distribution of the marker miRNAs in feeder cells. (B) Distribution of miRNAs in the Dlk1–Dio3 region. The protein coding genes and lncRNAs are marked as blocks. The miRNAs between genes are marked together. Type A, Type B and Conserved miRNAs are labeled in red, bule and grey, respectively. The marker miRNAs used for estimating the residual level of feeder cells are marked with a “#”. (C) Specifically expressed miRNAs or miRNA clusters in feeder cells compared to low and high passaged hECSs. The miRNA expression level (RPM) was normalized by log2. The miRNAs in the Dlk1-Dio3 region are marked in red, and miRNAs in other regions are marked in grey.


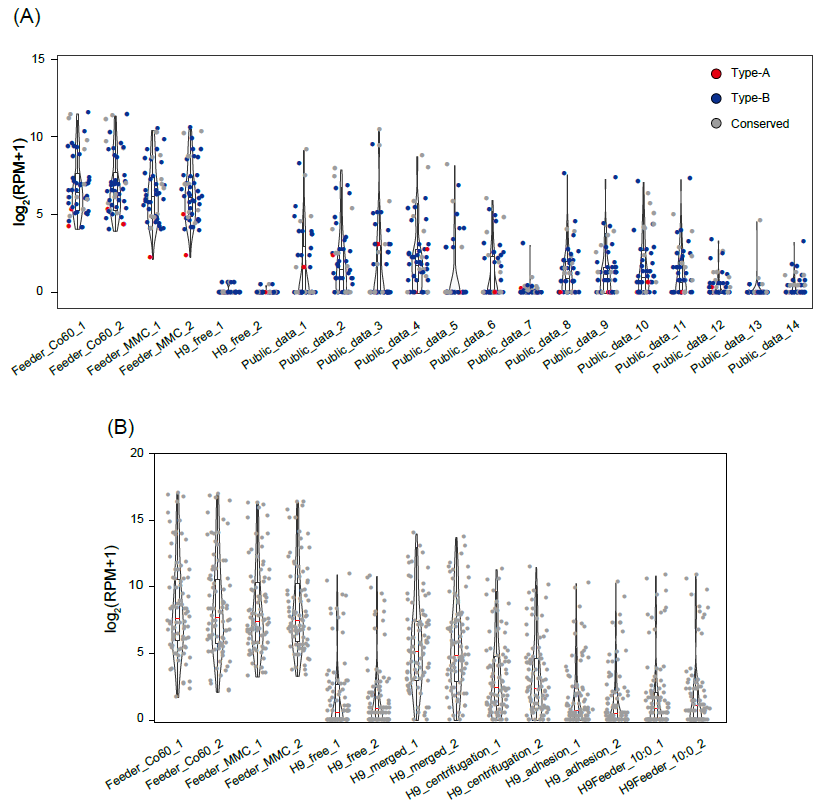


Figure S3. **Detection of miRNAs from residual feeder cells.**

(A) Levels of feeder cell specific miRNA residues in published miRNA-Seq datasets. (B) Conserved miRNAs between human and mouse whose expression levels might be influenced by reads derived from residual mouse feeder cells. The median value is marked in red.

**Supplementary Tables**

Table S1. **Public datasets used in this study.**

| Data types | Cell types | Accession IDs |
| --- | --- | --- |
| RNA-Seq | hESC cultured in feeder-free | SRR10409147, SRR10409148  SRR10409149, SRR10409150  SRR10409151, SRR10409152  SRR13820467, SRR13820468  SRR13820469 |
|  | Primed hESC cultured in feeder | SRR10409141, SRR10409142  SRR10409143, SRR10409144  SRR10409145, SRR10409146  SRR5125038, SRR5125039  SRR5125040, SRR5125047  SRR5125048, SRR5125049 |
|  | Primed hiPSC cultured in feeder | SRR10409159, SRR10409160  SRR10409161, SRR10409162  SRR10409163, SRR10409164 |
|  | Naïve hESC cultured in feeder | SRR10857233, SRR10857234  SRR13820455, SRR13820456  SRR5125041, SRR5125042  SRR5125043, SRR5125044  SRR5125045, SRR5125046  SRR5125050, SRR5125051  SRR5125052, SRR5125053  SRR5125054, SRR5125055  SRR8983632, SRR8983633 |
|  | mESC | SRR8146872, SRR8146873 |
|  | MEF | SRR8146876, SRR8146877  SRR8146878 |
|  | Hepatocyte | SRR8146882, SRR8146883  SRR8146884 |
| miRNA-Seq | mESC | SRR1042095, SRR1042096, SRR1042097 |
|  | MEF | SRR1042098, SRR1042099, SRR1042100 |
|  | hESC cultured in feeder | SRR1988287, SRR1988288, SRR1988289, SRR1988290,  SRR1203788, SRR493008, SRR493009, SRR493012, DRR003920***** |

*Six samples were gotten from DRR003920 using barcodes described in the Table S3 of ref. (An integrated expression atlas of miRNAs and their promoters in human and mouse).

Table S2. **Potentially influenced genes and miRNAs by the residual feeder cells.**

Table S3. **MiRNA type annotation for all the mouse and human miRNAs in miRbase.** The 4^th^ and 5^th^ column are other type RNAs found in the mouse and human genomes, respectively, overlapped with all possible locations of the miRNAs in the 1^st^ column.

Table S4. **List of the specifically expressed miRNAs or miRNA clusters in feeder cells.**

| **miRNA types** | **miRNAs/miRNA clusters** | | |
| --- | --- | --- | --- |
| Type A | *mmu-miR-3057-5p* | *mmu-miR-667-5p* |  |
| Type B | *mmu-miR-1188-5p* | *mmu-miR-337-5p* | *mmu-miR-666-5p* |
|  | *mmu-miR-1193-3p* | *mmu-miR-341-3p* | *mmu-miR-667-3p* |
|  | *mmu-miR-1198-5p* | *mmu-miR-344d-3p* | *mmu-miR-672-5p* |
|  | *mmu-miR-298-5p* | *mmu-miR-350-3p* | *mmu-miR-673-5p* |
|  | *mmu-miR-300-5p* | *mmu-miR-350-5p* | *mmu-miR-674-3p* |
|  | *mmu-miR-3068-3p* | *mmu-miR-351-5p* | *mmu-miR-679-5p* |
|  | *mmu-miR-3068-5p* | *mmu-miR-434-3p* | *mmu-miR-700-3p* |
|  | *mmu-miR-3102-3p* | *mmu-miR-5099* | *mmu-miR-701-5p* |
|  | *mmu-miR-322-3p* | *mmu-miR-540-3p* | *mmu-miR-770-3p* |
|  | *mmu-miR-329-3p* | *mmu-miR-540-5p* | *mmu-miR-872-3p* |
|  | *mmu-miR-337-3p* | *mmu-miR-666-3p* | *mmu-miR-872-5p* |
|  | *mmu-miR-1971//mmu-miR-1981-5p* | | |
|  | *mmu-miR-344-3p//mmu-miR-344b-3p//mmu-miR-344c-3p//mmu-miR-344e-3p//mmu-miR-344h-3p* | | |
| Conserved | *mmu-let-7d-3p___hsa-let-7d-3p* | | |
|  | *mmu-let-7i-3p___hsa-let-7i-3p* | | |
|  | *mmu-miR-127-5p___hsa-miR-127-5p* | | |
|  | *mmu-miR-196a-2-3p___hsa-miR-196a-3p* | | |
|  | *mmu-miR-214-5p___hsa-miR-214-5p* | | |
|  | *mmu-miR-223-3p___hsa-miR-223-3p* | | |
|  | *mmu-miR-615-3p___hsa-miR-615-3p* | | |
|  | *mmu-miR-615-5p___hsa-miR-615-5p* | | |
|  | *mmu-miR-665-3p___hsa-miR-665* | | |
|  | *mmu-miR-10a-3p//mmu-miR-10b-3p___hsa-miR-10a-3p//hsa-miR-10b-3p* | | |
|  | *mmu-miR-196a-5p//mmu-miR-196b-5p___hsa-miR-196a-5p//hsa-miR-196b-5p* | | |
